# The Isoforms of Ral Guanine Nucleotide Dissociation Stimulator (RalGDS) in LLC-PK1 Cells

**DOI:** 10.3390/cimb47070566

**Published:** 2025-07-18

**Authors:** Jingze Song, Na Li, Xinze Dong, Jianping Xie, Hongqiao Lai, Hengzhi Zhu, Kongwang He, Libin Wen, Sizhu Suolang, Qi Xiao

**Affiliations:** 1 Institute of Veterinary Medicine, Jiangsu Academy of Agricultural Sciences, Nanjing 210014, China; sjz18239127655@163.com (J.S.); lnna0115@163.com (N.L.); 15351755808@163.com (X.D.); 20100997@jaas.ac.cn (J.X.); qizhu7553@gmail.com (H.L.); paopao4545abc@163.com (H.Z.); kwh2003@263.net (K.H.); 2 Key Laboratory of Veterinary Biological Engineering and Technology, Ministry of Agriculture and Rural Affairs, Nanjing 210014, China; 3 Jiangsu Co-Innovation Center for Prevention and Control of Important Animal Infections Diseases and Zoonoses, Yangzhou University, Yangzhou 225009, China; 4 Jiangsu Key Laboratory for Food Quality and Safety—State Key Laboratory Cultivation Base of Ministry of Science and Technology, Nanjing 210014, China; 5 College of Animal Science, Tibet Agricultural and Animal Husbandry University, Provincial Key Laboratory of Tibet Plateau Animal Epidemic Disease Research, Linzhi 860000, China; xzslsz@163.com

**Keywords:** RalGDS, isoform, LLC-PK1 cells, frameshift mutation, molecular evolution

## Abstract

This study investigated the isoforms of porcine-origin Ral guanine nucleotide dissociation stimulator (RalGDS) in LLC-PK1 cells using reverse transcription-polymerase chain reaction (RT-PCR) and sequencing. Through segmented amplification, sequence assembly, and comparative genomics analysis, seven RalGDS isoforms were identified, characterized by insertions, deletions, and frameshift mutations. These genetic variations may significantly alter RalGDS’s protein structure and function, potentially impacting its role in Ral GTPase-mediated signaling pathways. This work provides foundational insights into the genetic diversity of porcine RalGDS and its implications for porcine physiology and economically significant traits.

## 1. Introduction

Ral guanine nucleotide dissociation stimulator (RalGDS), a member of the Ras-associate guanine nucleotide exchange factor (GEF) family, activates Ral GTPases by catalyzing GDP-to-GTP exchange. As a critical mediator downstream of Ras and Rap1 signaling pathways, RalGDS regulates cellular processes including proliferation, differentiation, and apoptosis. Structurally, RalGDS family members (RalGDS, RGL, RGL2/Rlf, and RGL3) share conserved domains: an N-terminal Ras Exchange Motif (REM), a central CDC25 homology domain, and a C-terminal Ras Binding Domain (RBD) [1,2,3]. RalGDS exemplifies how GEFs spatially and temporally control GTPase networks, making it a pivotal node in both physiology and disease. Its structural plasticity and context-dependent regulation remain active areas of investigation.

The RalGDS gene was initially cloned from a mouse cDNA library and has subsequently been identified in humans, cattle, zebrafish, and *Echinococcus granulosus* [3,4,5,6,7]. Notably, extensive alternative splicing generates numerous isoforms of this gene across species, including five transcript variants in humans and eleven in mice. In pigs (*Sus scrofa*), although the RalGDS sequence remains unannotated in public databases, in silico analysis of the porcine genome predicts at least twelve splice variants. Isoforms of RalGDS may confer structural and functional divergence, potentially influencing traits such as growth, disease resistance, and metabolic efficiency [8,9,10]. Currently, research reports on porcine RalGDS are scarce. The research indicates RalGDS as a potential biomarker for predicting IL12 production in pigs and reveals interaction between the host RalGDS protein and certain viruses, such as porcine circovirus-like virus P1 (the smallest known genome virus infecting animals) [11,12]. However, systematic characterization of porcine RalGDS isoforms is lacking.

Compared to in vivo experimentation, in vitro studies offer advantages including precise control over experimental conditions, operational ease, improved experimental consistency, elimination of animal use, and the potential to partially reflect in vivo outcomes. Porcine circovirus-like virus P1 can replicate in specific cell lines, including porcine kidney cell lines. Studies using porcine kidney cell models have demonstrated that porcine circovirus-like virus P1 inhibits the Wnt signaling pathway and activates the pancreatic secretion pathway, providing insights into its molecular pathogenesis [13,14,15]. The primary objective of this study was to determine the complete open reading frame (ORF) sequence of RalGDS in vitro, establishing a foundation for investigating how RalGDS-viral protein interactions affect viral replication, while simultaneously evaluating the potential utility of RalGDS for molecular-marker-assisted breeding. Here, we amplified the full-length RalGDS ORF from LLC-PK1 cells, a porcine renal proximal tubule-derived cell line, and identified seven novel isoforms. Therefore, this work advances our understanding of RalGDS genetic diversity in pigs and its potential biological significance.

## 2. Materials and Methods

### 2.1. Cells

LLC-PK1 cells were maintained in high-glucose Dulbecco’s Modified Eagle Medium (DMEM) (Gibco, Waltham, MA, USA), supplemented with 7% fetal bovine serum (FBS, Invitrogen, Waltham, MA, USA) and 1% penicillin/streptomycin at 37 °C in a humidified 5% CO_2_ incubator.

### 2.2. RT-PCR, DNA Cloning, and Sequence Assembly

Total RNA was extracted from confluent monolayers of LLC-PK1 cells using the QIAamp RNA Mini Kit (Qiagen, Hilden, Germany) according to the manufacturer’s instructions, followed by DNase treatment to remove contaminating genomic DNA. Reverse transcription was performed using 1 µg of total RNA with the HiFiScript gDNA Removal RT MasterMix Kit (Cowin Biotech Co., Ltd., Taizhou, China). To determine the full RalGDS ORF, primers were designed based on the twelve predicted RalGDS sequences (X1 (GenBank accession No. XM021070934), X2 (XM021070943), X3 (XM021070944), X4 (XM021070948), X5 (XM021070950), X6 (XM021070953), X7 (XM021070956), X8 (XM021070958), X9 (XM021070962), X10 (XR002338067), X11 (XR002338069), and X12 (XR002338070)) to generate overlapping fragments. Additional primers (primers 8 and 9) were synthesized based on newly amplified RalGDS sequences for verification (Table 1). PCR was conducted to amplify each cDNA fragment from the RT product using 2X Taq High-Fidelity Master Mix (Tsingke Biotech Co., Ltd., Nanjing, China) according to the manufacturer’s protocol. The PCR reaction was performed at 94 °C for 5 min, followed by 30 cycles of denaturation at 94 °C for 30 s, annealing at 58 °C for 30 s, and extension at 72 °C for 30 s, with a final extension at 72 °C for 5 min. To circumvent inaccuracies in terminal sequences inherent to direct sequencing of raw PCR products, each PCR amplicon was gel-purified, then cloned into the pUC-Blunt Zero cloning vector (Sangon Biotech, Shanghai, China), subsequently transformed into DH5α E. coli, and finally sequenced bidirectionally. At least five clones for each PCR product were sequenced. To ensure sequence reliability, each clone was sequenced in triplicate. Sequence assembly was performed using the DNAMAN software (version 9, Lynnon Biosoft, San Ramon, CA, USA).

The ten complete RalGDS mRNA sequences obtained in this study were deposited into the GenBank database under accession numbers PV013880–PV013882 and PV137743–PV137749.

### 2.3. Multiple-Sequence Alignments and Phylogenetic Analyses

The 22 near-full-length RalGDS sequences, including twelve predicted porcine RalGDS genes and ten sequences identified in this study, were used in sequence alignments and phylogenetic analyses. Sequence alignments were performed using DNAMAN software. Phylogenetic trees were constructed via the neighbor-joining method implemented in MEGA 7 software. Bootstrap analysis was computed with 1000 replicates to assess the reliability of each internal node, expressed as percentage values.

### 2.4. Physicochemical Analysis of RalGDS Proteins and Prediction of Global N6-Methyladenosine (m^6^A) Sites in RalGDS mRNA

Physicochemical properties and signal peptide prediction analyses were performed using ExPASy (ProtParam—SIB Swiss Institute of Bioinformatics|Expasy) (https://www.expasy.org/, accessed on 20 February 2025) and the SignalP 5.0 Server (SignalP 5.0—DTU Health Tech—Bioinformatic Services). Secondary structural analyses of the protein were performed using the online website Prab (https://npsa-prabi.ibcp.fr/, accessed on 26 February 2025). The prediction of m^6^A sites in RalGDS was implemented via the online server SRAMP (www.cuilab.cn/sramp/, accessed on 26 April 2025).

## 3. Results

### 3.1. Complete Genomic Characterization of RalGDS

**Figure 1:**
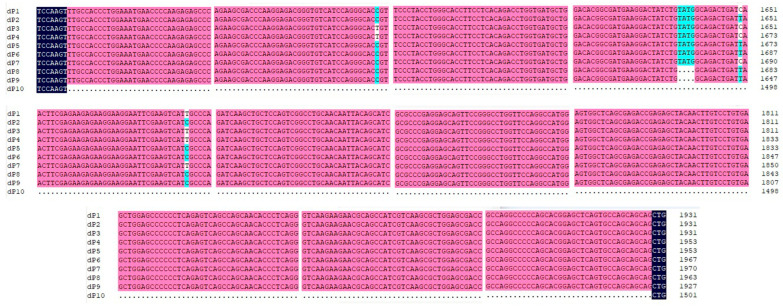
Alignment of nucleotide sequences of the variable region among ten RalGDS genes in this study. Dots represent the deletion mutation of 430 nucleotide sequences in RalGDS dP10 and 4 nt in RalGDS dP8 and dP9 compared to other RalGDS genes. Black, pink and blue represent nucleotide homology levels of 100%, 75% or higher and 50% or higher, respectively.

Ten distinct RalGDS sequences (dP1–dP10) were identified. The results indicated that the lengths of the mRNA sequences of RalGDS dP1, dP2, dP3, dP4, dP5, dP6, dP7, dP8, dP9, and dP10 were 3222, 3222, 3222, 3244, 3244, 3258, 3261, 3254, 3218, and 2792 nucleotides (nt), respectively. The 5′ untranslated regions (UTRs) of dP4 and dP5 were identical at 82 nt, while the 3′ UTRs of all dP sequences were identical at 599 nt. Compared to the predicted RalGDS sequences, the key features of the ten sequences obtained in this study were as follows: a contiguous 4-nucleotide deletion in dP8 and dP9; a contiguous 430-nucleotide deletion in dP10; and a single-base insertion in dP4 and dP5 (Figure 1).

**Figure 2:**
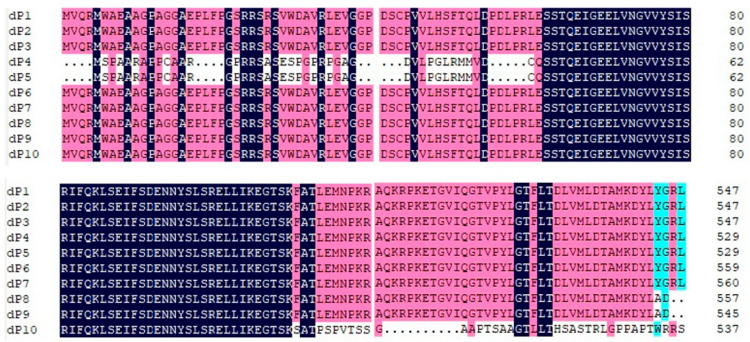
Alignment of amino acid sequences of the hypervariable region among four RalGDS genes. The N-terminal regions of dP4 and dP5, and the C-terminal domain of dP8, dP9, and dP10 demonstrate significant divergence in their amino acid sequences when compared to other members of the RALGDS family. Black, pink and blue represent amino acid homology levels of 100%, 75% or higher and 50% or higher, respectively.

The RalGDS sequences dP1, dP2, and dP3 each contain a long ORF of 2616 nt, encoding proteins of 872 amino acids (aa). dP4 and dP5 encode proteins of 854 aa. dP6 and dP7 encode proteins of 884 aa and 885 aa, respectively. In contrast, dP8, dP9, and dP10, dP8, dP9, and dP10, harboring frameshift mutations within their ORFs, encode significantly shorter proteins of 557 aa, 545 aa, and 538 aa, respectively. Furthermore, the 38-amino-acid sequence at the C-terminus of the dP10 protein exhibits extremely low homology compared to other RalGDS proteins (Figure 2).

The ten RalGDS genes shared 82.84–99.97% identity at the nucleotide level and 51.83–100.0% identity at the amino acid level. A subsequent comparison analysis with the twelve predicted *Sus scrofa* RalGDS sequences showed high divergence, ranging from 33.47% nucleotide identity between dP10 and X11 to 91.42% between dP7 and X1. The amino acid identity ranged from 50.34% between dP10 and X7 to 100.00% (Table 2).

The five known *Homo sapiens* RalGDS isoforms shared mRNA sequence identities ranging from 94.51% to 99.03%, and protein sequence identities ranging from 92.01% to 98.69%. Compared to the ten porcine RalGDS isoforms identified here, the sequence identities ranged from 62.83% to 76.41% at the mRNA level and 42.01% to 86.78% at the amino acid level.

The eleven known *Mus musculus* RalGDS isoforms exhibited mRNA sequence identities of 87.73–99.92% and protein sequence identities of 83.91–99.88%. Compared to the ten porcine RalGDS isoforms, the sequence identities showed mRNA identities of 59.43–74.42% and protein identities of 41.14–86.45%.

### 3.2. Phylogenetic Analysis

**Figure 3:**
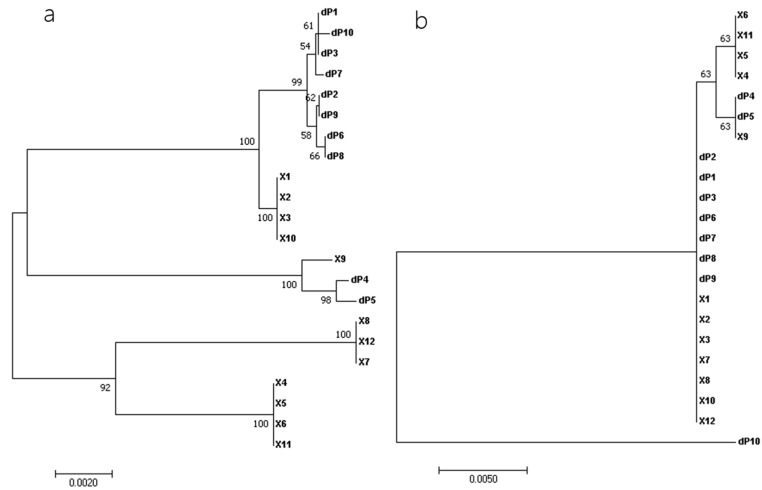
Phylogenetic analyses with the nucleotide sequences (**a**) and amino acid sequences (**b**) of the full-length RalGDS strains. Multiple-sequence alignments were performed using the ClustalW program implemented in MEGA 7, and phylogenetic trees were constructed using the neighbor-joining method. Bootstrap values > 50% (1000 replicates) of NJ analysis are shown above the branches.

Phylogenetic relationships among the 22 near-full-length RalGDS sequences were estimated. The nucleotide sequence-based phylogenetic tree demonstrated that dP1, dP2, dP3, dP6, dP7, dP8, dP9, and dP10 clustered together and were closely related to X1, X2, X3, and X10, while dP4 and dP5 aligned with X9, distinct from two lineages: X8, X12, X7, and X4, X5, X6, X11. In contrast, the phylogenetic tree based on amino acid sequences differed from that based on nucleotide sequences, revealing that dP1, dP2, dP3, dP6, dP7, dP8, and dP9 clustered with X1, X2, X3, X7, X8, X10, and X12; dP4 and dP5 were closely related to X9, X4, X5, X6, and X11. Notably, dP10 formed a distinct branch, highlighting its evolutionary divergence (Figure 3).

### 3.3. Physicochemical Properties, Secondary Structure Analysis, and m6A Sites’ Prediction

The physicochemical properties and predicted secondary structures of the ten RalGDS proteins are summarized in Table 3. Their amino acid lengths ranged from 538 aa (dP10) to 885 aa (dP7). The molecular weights varied from 58,657.73 Da (dP10) to 97,399.31 Da (dP7). The isoelectric points (pI) of the ten RalGDS proteins spanned from 5.29 to 5.96, indicating that all ten proteins are acidic. The highest aliphatic index was recorded for dP9 (89.69), whereas dP4 and dP5 exhibited the lowest (80.90). The GRAVY (Grand Average of Hydropathicity) values for all ten RalGDS proteins were below zero, indicating hydrophilic character. The instability index ranged from 45.95 to 53.19, classifying all ten RalGDS proteins as theoretically unstable.

No signal peptides (Sec/SPI) were predicted among the ten proteins. Secondary structure prediction indicated that α-helix (37.47–48.65%) and random coil (34.83–46.60%) constituted the largest proportions, followed by extended strand (9.85–12.10%) and β-turn (3.86–5.39%). The secondary structure composition of dP8, dP9, and dP10 differed significantly from that of dP1-dP7, characterized by increased proportions of α-helix and β-turn and decreased proportions of extended strand and random coil.

SRAMP prediction identified 15 high-probability m^6^A sites (>0.50) in dP1, dP2, dP3, dP4, dP5, dP6, dP8, and dP10 mRNA; 14 sites in dP7 mRNA; and 16 sites in dP9 mRNA. Notably, RalGDS dP6, dP7, and dP8 mRNAs exhibited six m^6^A sites each with probability thresholds between 0.80 and 0.90, a quantity twice that observed in the other mRNAs.

## 4. Discussion

This study systematically characterizes RalGDS isoforms in porcine LLC-PK1 cells, identifying seven novel variants with potential functional implications. Based on 5′ terminal sequence homology, the twelve predicted RalGDS genes were classified into four categories: Category 1: X1, X2, X3, X10; Category 2: X7, X8, X12; Category 3: X4, X5, X6, X11; and Category 4: X9. We designed primers for these four categories of genes, amplified them in segments, and finally designed primers based on the amplified sequences to amplify longer fragments for verification.

In this study, ten RalGDS mRNAs (dP1–dP10) were identified in LLC-PK1 cells. Sequence homology analysis revealed that dP1, dP2, and dP3 are closely related to the predicted RalGDS X3; dP6 is closely related to X2; and dP7 is closely related to X1. Similarly, dP4 and dP5 are closely related to the predicted X9. Notably, the single-base insertion in dP4 and dP5 results in an extended N-terminal region. The observed frameshift mutations (a 4 nt deletion in dP8 and dP9, and 430 nt deletion in dP10) are predicted to disrupt the RalGDS domain architecture, particularly affecting the CDC25 and RBD domains essential for GTPase activation. RalGDS exhibits GDP-to-GTP exchange activity, which is dependent on the CDC25 homology domain located in the central region of the protein, and the ability of RalGDS to bind Ras and Ras-related proteins depends on the RBD located in the C-terminal region of the protein [1]. Consequently, structural alterations such as deletions within these domains may impair RalGDS-mediated signaling.

The results of the phylogenetic tree based on amino acid sequences showed that RalGDS genes were divided into three categories: Cluster 1 comprised dP1, dP2, dP3, dP6, dP7, dP8, and dP9; Cluster 2 comprised dP4 and dP5; and dP10 formed a distinct evolutionary branch (Cluster 3).

Protein physicochemical properties are critical for understanding function [16]. Our results indicate that most members of the RalGDS gene family, including dP1, dP2, dP3, dP4, dP5, dP6, and dP7, which exhibit high homologous conservation in their amino acid coding sequences, possess similar values for the protein instability index, aliphatic index, and secondary structure. This contrasts significantly with dP8, dP9, and dP10, where deletions causing frameshift mutations result in divergent properties. The tertiary structure refers to the three-dimensional conformation of a protein molecule in its native folded state, which is formed by further coiling and folding based on the secondary structure. Differences in secondary structure led to variations in the protein’s tertiary structure, thereby affecting its biological function. Meanwhile, the physicochemical properties of proteins arise from the interplay between their structures (ranging from primary sequences to higher-order conformations) and their environment; variations in these properties manifest molecular-level reflections of functional diversification, evolutionary adaptation, and regulatory mechanisms. Therefore, the biological functions of RalGDS proteins dP1–dP7 are likely distinct from those of dP8–dP10.

The RalGDS gene exhibits multiple transcript variants across species. For instance, human isoforms encode proteins ranging from 859 to 914 amino acids, while murine isoforms encode proteins of 782 to 907 amino acids. Despite variations in length, their C-terminal sequences demonstrate significant conservation. Notably, the porcine RalGDS dP10 identified here lacks C-terminal homology with other porcine RalGDS isoforms. The significant phylogenetic divergence of dP10 underscores its unique evolutionary trajectory. Its low homology to other variants (<51% amino acid identity) and distinct physicochemical properties (e.g., reduced molecular weight, altered secondary structure) suggest potential neofunctionalization or subfunctionalization. Further studies should investigate whether dP10 is expressed in vivo in pigs and assess if it retains GTPase activation capacity or acquires novel roles, such as in porcine renal physiology. Numerous studies have established associations between RalGDS isoforms and the pathogenesis of various diseases, including skin cancer, breast cancer, and cardiovascular diseases [17,18,19,20,21,22,23,24]. Given that RalGDS dP10 was detectable exclusively in porcine kidney cell lines and not in other cell lines or healthy porcine tissues, and considering the spontaneously immortalized nature of LLC-PK1 cells (sharing characteristics with cancer cells), dP10 warrants investigation as a potential diagnostic biomarker for porcine renal cell carcinoma.

N^6^-methyladenosine (m^6^A) is the most prevalent internal mRNA modification, influencing diverse RNA processes including subcellular localization, splicing, stability, and conformation [25]. Differences in the number of m6A sites among the RalGDS isoforms identified in this study could potentially modulate its biological functions.

While this study focused on a cell line model, future work should validate the presence and function of these isoforms in vivo and assess their potential association with economically relevant traits such as growth efficiency or disease susceptibility.

## 5. Conclusions

We report the first identification of seven RalGDS isoforms in LLC-PK1 cells, including variants characterized by frameshift mutations and insertions predicted to alter protein function. These findings enrich the porcine genomic database and establish a molecular framework for investigating the role of RalGDS in porcine physiology and economically significant traits. Future research should prioritize in vivo validation and mechanistic studies to harness RalGDS diversity, including epigenetic factors such as promoter methylation and its potential regulatory effects, to apply these findings into practical applications for animal genetic improvement programs.

## Figures and Tables

**Table 1:** PCR primers used for amplification of the porcine RalGDS gene.

Primers	Primer Sequences	Target Genes	PCR Product Size
1	F26-CCGTCCATGGTGCAGCGCATGTG; R721-GGTCGGAGCCGGGCATGTTGAGC	X1, X2, X3, X10	814
2	F82-GCGTTCATGCTGGTGGTCC; R742-CTAGCTCTGGGGCTGGTTTC	X7, X8, X12	661
3	F4341-ACCGGAAGAGGATGTGCAAC; R5220-CACTGGACTGGGTGCTAGAAG	X4, X5, X6, X11	880
4	F234-CGGCTCAAACAATGGGATCAG; R1027-TCTAGCTCTGGGGCTGGTTT	X9	794
5	F244-GAGTCGGCCCTGAACCTGTATGA; R1517-GGTACCCTGGATGACACCCGTCT	All	1274
6	F979-GGAGCTGGCTCTGTCGCAAAG; R2334-CGGATGATGCAGCAGTCGC	All	1356
7	F1833-TGGAGTGGCTCAGCGAGACTG; R3287-TGAGCGTGGTCTGCGAAGAG	All	1455
8	F136-CCGGTGGTGCTGCACAGCTTCA; R2065-TCTCCTGGCCATCGGGGGACTC	X1, X2, X3, X10	1951
9	F193-TCCACACAGGAGATTGGCGAGG; R2429-CAGCTTGCGATCTTCCGAAATG	X9	2258

**Table 2:** Identity comparison of the ten RalGDS genes in this study to twelve reference *Sus scrofa* RalGDS genes.

Reference Gene (Length)	dP1	dP2	dP3	dP4	dP5	dP6	dP7	dP8	dP9	dP10
X1 (3554 bp)	90.35	90.35	90.35	87.75	87.75	91.33	91.42	91.22	90.24	78.33
X2 (3551 bp)	90.43	90.43	90.43	87.83	87.83	91.41	91.33	91.30	90.31	78.40
X3 (3514 bp)	91.38	91.38	91.38	88.70	88.70	90.42	90.35	90.31	91.26	79.23
X4 (7900 bp)	39.36	39.36	39.36	39.42	39.42	39.80	39.84	39.75	39.31	33.95
X5 (7897 bp)	39.37	39.37	39.37	39.44	39.44	39.82	39.80	39.76	39.32	33.97
X6 (7484 bp)	41.54	41.54	41.54	41.61	41.61	41.33	41.32	41.28	41.49	35.84
X7 (3460 bp)	87.73	87.73	87.73	86.68	86.68	88.73	88.65	88.61	87.62	75.64
X8 (3424 bp)	88.64	88.64	88.64	87.57	87.57	87.71	87.63	87.59	88.52	76.42
X9 (3744 bp)	83.00	83.00	83.00	86.28	86.28	82.18	82.12	82.07	82.89	71.64
X10 (3477 bp)	88.08	88.08	88.08	85.56	85.56	89.06	89.15	88.95	87.96	77.65
X11 (7783 bp)	38.59	38.59	38.59	38.61	38.61	38.41	38.39	38.35	38.54	33.47
X12 (3345 bp)	86.38	86.38	86.38	85.32	85.32	85.47	85.39	85.35	86.26	75.84
X1 (885 aa)	98.53	98.53	98.53	92.54	92.54	99.89	100.00	62.71	61.24	56.95
X2 (884 aa)	98.64	98.64	98.64	93.21	93.21	100.00	99.77	62.78	61.43	57.01
X3 (872 aa)	100.00	100.00	100.00	94.50	94.50	98.64	98.42	61.43	62.27	57.80
X4 (858 aa)	92.09	92.09	92.09	94.46	94.46	94.01	93.67	56.84	54.92	51.07
X5 (857 aa)	92.76	92.76	92.76	94.57	94.57	94.12	93.45	56.90	55.09	51.13
X6 (845 aa)	94.04	94.04	94.04	95.90	95.90	92.76	92.09	55.54	55.85	51.83
X7 (835 aa)	91.97	91.97	91.97	93.88	93.88	93.33	93.11	56.11	54.75	50.34
X8 (823 aa)	93.23	93.23	93.23	95.20	95.20	91.97	91.75	54.75	55.50	51.03
X9 (817 aa)	93.00	93.00	93.00	95.67	95.67	91.74	91.53	54.52	55.28	50.80
X10 (762 aa)	78.85	78.85	78.85	74.12	74.12	80.20	80.43	72.74	71.04	66.06
X11 (722 aa)	73.74	73.74	73.74	76.23	76.23	72.75	72.55	64.44	64.93	60.27
X12 (700 aa)	73.97	73.97	73.97	75.53	75.53	72.41	72.22	63.52	64.53	59.33

**Table 3:** Protein properties and structure of RalGDS in LLC-PK1 cells.

RalGDS Strain	Protein Length (AA)	MW(Da)	pI	Instability Index	Aliphatic Index	GRAVY	Secondary Structure Prediction (SOPMA)			
							Alpha Helix	Extended Strand	Beta Turn	Random Coil
dP1, dP2, dP3	872	96030.83	5.71	51.91	82.24	−0.338	38.30	11.70	4.47	45.53
dP4, dP5	854	93789.38	5.96	53.19	80.90	−0.351	37.47	12.06	3.86	46.60
dP6	884	97312.24	5.77	51.82	81.79	−0.340	38.57	12.10	4.75	44.57
dP7	885	97399.31	5.77	52.21	81.69	−0.341	38.31	12.09	4.63	44.97
dP8	557	61323.00	5.35	45.95	88.82	−0.226	48.65	11.13	5.39	34.83
dP9	545	60041.59	5.29	45.98	89.69	−0.219	48.44	10.46	4.95	36.15
dP10	538	58657.73	5.50	49.70	87.43	−0.225	45.72	9.85	4.46	39.96

## Data Availability

All sequencing data are available from the GenBank database on the NCBI website.
